# A Complex Case of Extensive Systemic Amyloidosis With Underlying Monoclonal Gammopathy

**DOI:** 10.7759/cureus.43753

**Published:** 2023-08-19

**Authors:** Faisal Syed, Mubariz A Hassan, Jeswin Joy, Oluwatayo J Awolumate, Uzoamaka Nwaogwugw

**Affiliations:** 1 Internal Medicine, Howard University Hospital, Washington, USA; 2 Nephrology, Howard University Hospital, Washington, USA

**Keywords:** systemic, chemotherapy, embolism, gammopathy, amyloidosis

## Abstract

Systemic amyloid light chain, or primary amyloidosis (AL amyloidosis), is a serious medical condition that leads to the deposition of abnormal proteins called amyloid fibrils in various organs of the body. AL amyloidosis can present with different symptoms, which can make diagnosis challenging. This case report presents a clinical scenario of a 53-year-old female patient who had come in for shortness of breath and lower extremity swelling and was found to have acute on chronic pulmonary embolism. The patient had a history of systemic amyloidosis diagnosed with a kidney and duodenal biopsy. She also had a bone marrow biopsy done and was found to have IgG monoclonal gammopathy. Throughout the hospital course, patients required cautious diuretic use given the worsening kidney function. She was given intravenous anticoagulation initially and later switched to oral medication on discharge. Due to the aggressive nature of amyloidosis, a decision was made to start the patient on chemotherapy in an outpatient setting. This case presents an interesting scenario of systemic amyloidosis with concomitant monoclonal gammopathy that was complicated by acute pulmonary embolism. The case is important as it shows the different levels of amyloidosis and teaches us the benefit of taking a multidisciplinary approach to making a concrete plan for patients with advanced amyloidosis disease.

## Introduction

Systemic amyloidosis is a group of disorders characterized by the accumulation of insoluble proteins in tissues. The most common form of systemic amyloidosis is light-chain amyloidosis (AL), which results from the accumulation of misfolded immunoglobulins. Different organs of the body are involved, including the heart, kidney, liver, peripheral nerves, and gastrointestinal tract. It is usually a progressive disease, and mortality is often predicted by cardiac, renal, or autonomic dysfunction. Treatment options include targeting the underlying plasma cell dyscrasia to prevent the production of further amyloidogenic proteins [1]. Here we have a clinical case of a 53-year-old female who initially had monoclonal gammopathy diagnosed on serum and urine immunofixation (IFE) and was later found to have AL amyloidosis. Our patient was diagnosed with renal and GI amyloidosis after a biopsy. Cardiac amyloidosis was ruled out with a cardiac magnetic resonance imaging (MRI) scan. The aim of this case report is to unravel the complexities of extensive systemic amyloidosis and underscore the need for a comprehensive approach to management.

## Case presentation

A 53-year-old female with a past medical history of hypertension, chronic kidney disease stage G3B-IV/A3 with rapid progression to stage 4, systemic amyloidosis (renal, gastrointestinal), IgG lambda monoclonal gammopathy, nephrotic syndrome with chronic anasarca, and previous bilateral pulmonary embolism presented to the emergency department (ED) due to worsening shortness of breath and bilateral leg swelling. The patient was previously diagnosed with renal amyloidosis by kidney biopsy due to worsening nephrotic range proteinuria. She also had an upper endoscopy done, which confirmed small intestinal amyloidosis. Previous workups included total protein electrophoresis with immunofixation (IFE) (Table 1), along with serum (Table 2) and urine IFE, which showed increased levels and an abnormal band of IgG lambda, respectively. A bone marrow biopsy was done after this and showed a myeloid and erythroid cell ratio of 2-3:1. Scattered plasma cells were also seen, accounting for 8%-10% of total marrow cellularity.

**Table 1:** Total protein electrophoresis with immunofixation analysis

Type	Level	Reference Range
Total Protein	3.5g/dl (Low)	6.1-8.1
Albumin	1.1g/dl (Low)	3.8-4.8
Alpha 1 Globulin	0.3g/dl	0.2-0.3
Alpha 2 Globulin	1.2g/dl (High)	0.5-0.9
Beta 1 Globulin	0.2g/dl (Low)	0.4-0.6
Beta 2 Globulin	0.2g/dl	0.2-0.5
Gamma Globulin	0.6 L g/dl	0.8-1.7
Abnormal Protein Band 1	0.4 g/dl (High)	-

**Table 2:** Serum immunofixation analysis

Serum Immunofixation	Level	Reference range
Immunoglobulin A	81mg/dl	47-310
Immunoglobulin G	480mg/dl (Low)	600-1640
Immunoglobulin M	61mg/dl	50-300

**Figure 1:**
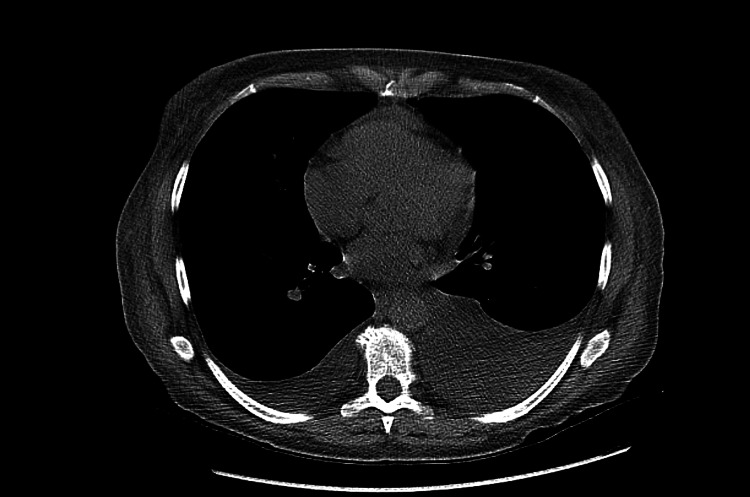
CT abdomen and pelvis with signs of pleural effusion

**Figure 2:**
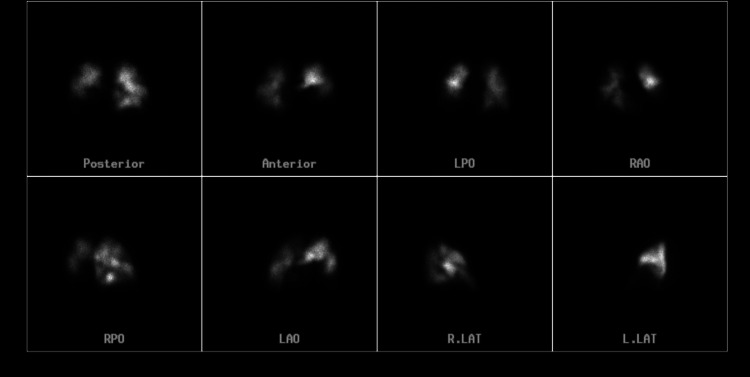
Ventilation-Perfusion (V/Q) scan confirming acute on chronic pulmonary embolism

On this admission, vitals in the emergency department showed a BP of 133/101 mmHg, a T of 98.4F, a pulse rate of 106/min, and oxygen saturation of 100% in room air. Initial labs were significant for elevated creatinine and white blood cell count (WBC), along with decreased levels of bicarbonate and albumin (Table 3). CT abdomen pelvis without contrast showed bilateral pleural effusions, pericardial fluid, ascites, and diffuse anasarca (Figure 1). The patient was admitted for worsening shortness of breath and severe anasarca. The patient was started on diuretics. The patient was also started on a heparin drip after a ventilation perfusion (V/Q) scan showed acute to chronic pulmonary embolism (Figure 2).

**Table 3:** Lab results

Basic Lab	Results	Reference Range
White Blood Cell	15.36	3.2-10.6x10^9^/L
Hemoglobin	9.3	14.6-17.8 g/dL
Platelet	418	177-406x109/L
Sodium	143	135-145 mEq/L
Potassium	4.4	3.5-5.1 mEq//L
Creatinine	3.88	0.6-1.2 mg/dL
Blood Urea Nitrogen	28	7-25 mg/dL
Albumin	1.69	3.2-5.5g/dl

Nephrology and oncology were consulted for further input. Diuresis was held per nephrology recommendations due to worsening kidney function and acidosis. Patients received intravenous (IV) and oral bicarbonate in the meantime. Oncology recommends induction chemotherapy in an outpatient setting, given the aggressive nature of amyloidosis. The patient also had a drop in hemoglobin (Hb) levels during the hospital course but refused a blood transfusion and instead preferred intravenous iron. Later on, the patient's acidosis and creatinine levels improved, and she was restarted on cautious diuretics. Her shortness of breath was significantly better, with a decrease in lower extremity swelling as well. She remained clinically stable and was discharged with outpatient follow-up with nephrology and oncology. The patient was continued on oral anticoagulation with apixaban. Post-discharge, the patient was followed in an outpatient setting. The patient had a chest port placed and was started on chemotherapy with CyBorD therapy (Cyclophosphamide, Bortezomib, and Dexamethasone), complemented with Daratumumab. The patient also had a cardiac magnetic resonance imaging (MRI) scan done, which ruled out cardiac amyloidosis. Nephrology follow-up is being continued, and the patient has remained compliant with all her medications.

## Discussion

Extensive systemic amyloidosis is characterized by the extracellular deposition of insoluble amyloid fibrils derived from various precursor proteins, leading to multisystem dysfunction [2]. Monoclonal gammopathy, in contrast, involves the clonal proliferation of plasma cells and the production of a monoclonal immunoglobulin, further worsening organ damage and dysfunction [3]. The interplay between these conditions poses significant clinical challenges, highlighting the need for a multidimensional treatment strategy.

Renal involvement is a prominent feature of systemic amyloidosis and plays a significant role in the clinical presentation of the patient. The deposition of amyloid fibrils within the renal parenchyma disrupts the normal architecture and function of the kidneys, leading to progressive renal impairment. Glomerular involvement is common, with amyloid deposits accumulating within the glomerular basement membrane, mesangial, and capillary walls. This results in glomerular sclerosis, thickening of the basement membrane, and narrowing of the capillary lumens, ultimately leading to proteinuria and nephrotic syndrome. Additionally, tubular dysfunction may occur, characterized by impaired reabsorption and concentration ability, leading to electrolyte imbalances, polyuria, and polydipsia. In advanced stages, amyloid deposition within the interstitium can cause interstitial fibrosis and tubular atrophy, further contributing to the decline in renal function. The renal manifestations of systemic amyloidosis highlight the importance of early detection and intervention to preserve renal function and mitigate the risk of renal failure. Close monitoring of renal parameters, such as proteinuria, serum creatinine, and estimated glomerular filtration rate, is crucial in managing patients with systemic amyloidosis and initiating timely therapeutic interventions to slow disease progression. Systemic amyloidosis can predispose individuals to thromboembolic events through various mechanisms. The deposition of amyloid fibrils within blood vessel walls can disrupt the normal structure and function of the vascular endothelium, promoting a pro-thrombotic state. The amyloid deposits can activate platelets, leading to platelet aggregation and the formation of thrombi [4].

Moreover, amyloid fibrils can interact with coagulation factors, such as fibrinogen and von Willebrand factor, impairing their normal regulation and promoting abnormal clot formation. Additionally, amyloidosis-induced organ dysfunction, particularly cardiac and renal involvement, can further contribute to thromboembolism. Cardiac amyloidosis can cause atrial fibrillation, leading to blood stasis within the atria and an increased risk of thrombus formation. Renal impairment in amyloidosis can result in proteinuria and a reduction in anticoagulant proteins, such as antithrombin III, further increasing the propensity for thrombosis. These various mechanisms collectively increase the risk of thromboembolic events in individuals with systemic amyloidosis [5].

The patient's manifestation of worsening shortness of breath and fluid overload underscores the cardiopulmonary implications commonly observed in amyloidosis. The infiltration of amyloid into the myocardium can result in restrictive cardiomyopathy, leading to diastolic dysfunction and heart failure [6]. Concurrent renal involvement may exacerbate fluid overload, resulting in anasarca and necessitating the administration of high doses of diuretics. Furthermore, the patient's severe anemia could be attributed to bone marrow infiltration by myeloma, compromised erythropoiesis, or gastrointestinal bleeding secondary to amyloid involvement [7].

To address both the underlying plasma cell dyscrasia and amyloidosis, the patient was initiated on a chemotherapy regimen comprising CyBorD plus Daratumumab. CyBorD, a well-established therapeutic combination in multiple myeloma, aims to suppress the abnormal plasma cell population through various mechanisms of action. Cyclophosphamide functions as an alkylating agent, impairing DNA synthesis and cell division. Bortezomib, a proteasome inhibitor, disrupts protein degradation pathways, ultimately inducing apoptosis in neoplastic plasma cells. Dexamethasone, a corticosteroid, possesses anti-inflammatory and immunosuppressive properties. The integration of Daratumumab, a monoclonal antibody targeting CD38, into the chemotherapy regimen further augments the anti-myeloma effect. CD38 exhibits high expression on plasma cells, and Daratumumab exerts its action through multiple mechanisms, including antibody-dependent cellular cytotoxicity and complement-dependent cytotoxicity, ultimately leading to the depletion of plasma cells [8].

The patient's concurrent anticoagulation therapy for pulmonary embolism introduces an added layer of complexity, as it poses a potential risk of bleeding complications during chemotherapy. The delicate balance between managing thrombotic events and minimizing bleeding risk necessitates close monitoring and personalized therapeutic decision-making.

## Conclusions

The case of extensive systemic amyloidosis accompanied by monoclonal gammopathy presents a captivating clinical scenario that requires a multidisciplinary approach. The incorporation of intensive chemotherapy aims to address the underlying plasma cell dyscrasia while simultaneously managing the systemic manifestations of amyloidosis. Consideration of concurrent comorbidities, such as pulmonary embolism requiring anticoagulation, should also be vigilantly monitored. The intricate interplay between these disease processes emphasizes the ongoing need for research and the development of targeted therapies to enhance management strategies for patients experiencing similar presentations.
